# Beyond Blame: Is India Ready for No-Fault Liability in Healthcare?

**DOI:** 10.7759/cureus.58111

**Published:** 2024-04-12

**Authors:** Satvik N Pai, Madhan Jeyaraman, Naveen Jeyaraman, Sankalp Yadav

**Affiliations:** 1 Orthopaedics, PES University Institute of Medical Sciences and Research, Bengaluru, IND; 2 Clinical Research, Virginia Tech India, Dr. MGR Educational and Research Institute, Chennai, IND; 3 Orthopaedics, ACS Medical College and Hospital, Dr. MGR Educational and Research Institute, Chennai, IND; 4 Medicine, Shri Madan Lal Khurana Chest Clinic, New Delhi, IND

**Keywords:** fault attribution, justice, healthcare, liability, no-fault

## Abstract

In contemporary healthcare systems, the pursuit of justice intertwines with fault attribution and liability determination. The exploration of no-fault liability as a potential alternative within India's healthcare landscape delves into its feasibility and implications. Drawing from international experiences, regulatory frameworks, and societal readiness, the complexities and potential benefits of adopting a no-fault liability system are elucidated. Perspectives from patients, healthcare providers, and broader societal stakeholders are considered, highlighting both the advantages and challenges associated with such a transition. Addressing disparities in access, legal reforms, and logistical hurdles underscores the groundwork necessary for potential adoption, signaling a potential paradigm shift toward equitable compensation and accountability within India's healthcare system.

## Editorial

In contemporary healthcare systems, the pursuit of justice intertwines with the complexities of fault attribution and liability determination. Amidst this intricate landscape, the concept of no-fault liability emerges as a compelling alternative, offering a paradigm shift from traditional fault-based approaches. Rooted in the principle of providing compensation to injured parties without the need to establish negligence, no-fault systems have garnered attention for their potential to streamline claims processes, enhance patient outcomes, and alleviate the adversarial nature of medical malpractice litigation. We seek to explore the feasibility and implications of adopting no-fault liability within the context of healthcare, with a particular focus on its potential implementation in India. As a country grappling with diverse healthcare challenges, ranging from patient safety concerns to issues of medical negligence, India stands at a critical juncture in evaluating alternative models of compensation and accountability. Through an examination of international experiences, regulatory frameworks, and societal readiness, this article aims to elucidate the readiness of India's healthcare ecosystem to embrace the transformative principles of no-fault liability.

What is ‘no-fault liability’ and why should we consider it?

No-fault liability is a legal concept where compensation is provided to individuals who have been harmed or injured without the need to prove that someone else was at fault or negligent. In the context of healthcare and medical negligence, it means that patients who suffer harm during medical treatment can receive compensation for their injuries without having to prove that the healthcare provider was negligent [1].

No-fault liability offers several advantages from multiple perspectives, making it a desirable framework in the context of healthcare. From the patient's standpoint, no-fault liability provides a streamlined process for receiving compensation in cases of medical harm without the need to endure lengthy and adversarial legal battles. It ensures that patients promptly receive the support they need for medical expenses, rehabilitation, and other necessary services, facilitating a smoother recovery process. For doctors, no-fault liability can alleviate the fear of facing litigation and reputational damage due to malpractice claims. It fosters an environment where healthcare providers can prioritize patient care without the constant threat of legal repercussions, enabling them to focus on delivering high-quality treatment and improving outcomes. Moreover, from the perspective of society as a whole, no-fault liability promotes transparency, accountability, and fairness within the healthcare system. By shifting the focus away from assigning blame and toward compensating injured parties, it helps build trust between patients and healthcare providers, enhancing overall confidence in the healthcare system. Additionally, it reduces the burden on the judicial system and healthcare resources, redirecting them toward more productive endeavors and ultimately contributing to the well-being of society.

If everyone is benefiting, why don’t we adopt it then?

While no-fault liability systems offer several advantages, they are not without drawbacks. One significant drawback is the potential reduction in accountability among healthcare providers. Without the requirement to prove negligence, some argue that no-fault systems may diminish the incentive for healthcare professionals to uphold rigorous standards of care, leading to complacency or laxity in practice [2]. There are concerns about sourcing the funds and the practical complexities of claim adjudication. A major roadblock is the financial sustainability of such systems. While they aim to streamline compensation processes and ensure prompt redress for injured parties, sourcing funds for compensation presents a formidable challenge. The collective funding required often involves contributions from government entities, healthcare providers, insurance companies, and sometimes patients, necessitating a delicate balance to ensure adequate and equitable funding without overburdening any single stakeholder. Moreover, the determination of genuine claims poses practical challenges. Without the need to establish fault, there is a risk of increased incidences of frivolous or exaggerated claims, potentially straining resources and undermining the integrity of the compensation system. Adjudicating the authenticity of claims becomes a complex endeavor, requiring robust mechanisms for investigation, documentation, and validation to distinguish between legitimate grievances and opportunistic claims. These practical challenges, coupled with the financial complexities of sourcing funds, underscore the intricacies and potential pitfalls of implementing a no-fault liability system in healthcare.

Has it been practiced in other countries and how has it fared?

Several countries have adopted variations of the no-fault liability system in healthcare, each with its own experiences and approaches to implementation. Sweden, New Zealand, and Denmark are among the pioneers in embracing this alternative framework for compensating medical injuries. Sweden's Patient Injury Act, established in 1975, provides compensation to patients for injuries resulting from medical treatment, emphasizing patient-centered care and swift resolution of claims [3]. Funding primarily comes from taxes and healthcare contributions. Similarly, Denmark operates a no-fault liability system overseen by the Patient Compensation Association, funded through contributions from healthcare providers and the state. New Zealand's Accident Compensation Corporation (ACC), established in 1974, covers a wide range of injuries, including those from medical treatment, funded through levies and subsidies [4]. In the year 2004 alone, the ACC processed over 250,000 claims related to medical injuries, demonstrating the system's accessibility and efficiency. Moreover, surveys of claimants have shown high levels of satisfaction with the process, with approximately 90% reporting satisfaction with the support received and the overall handling of their claims [4].

Overall, the general experience with no-fault liability in these countries has been positive. These systems are praised for their efficiency, accessibility, and ability to provide prompt compensation to injured parties without the need to establish fault. While each country may differ slightly in its approach to implementation and funding, the core principle remains consistent: compensating patients for medical injuries without the burden of proving negligence. Despite criticisms regarding the complexity of the claims process and the need for greater transparency and accountability, the overarching consensus is that no-fault liability systems have been effective in delivering compensation and support to those affected by medical harm.

However, despite the overall positive reception, challenges have emerged within the no-fault liability systems of these countries. Issues such as administrative complexities, delays in claim processing, and concerns regarding fairness and transparency have been reported [5]. These challenges underscore the need for ongoing refinement to ensure effectiveness while maintaining fairness, transparency, and efficiency.

Is India ready for this?

Assessing the readiness of India for a no-fault liability system in healthcare demands a comprehensive understanding of the unique context, mentality, thinking, and practices prevalent among Indian patients, healthcare providers, and the broader societal landscape. From the perspective of patients in India, the implementation of such a system could potentially offer streamlined compensation processes and expedited redress for medical injuries, alleviating the burden of lengthy legal battles. However, ensuring accessibility, fairness, and trust in the compensation process would be crucial, particularly for marginalized and vulnerable populations who may face barriers in navigating the healthcare system.

Similarly, Indian healthcare providers may perceive a no-fault liability system as a means to mitigate the fear of protracted litigation and reputational damage, enabling them to prioritize patient care [5]. However, challenges may arise in navigating administrative complexities and ensuring adherence to quality standards amidst varying healthcare practices. Providing adequate support, training, and guidance for healthcare professionals would be essential for the effective implementation and acceptance of such a system.

From the perspective of the judiciary, adopting a no-fault liability system would necessitate reforms to legal frameworks and procedures to accommodate the shift away from fault-based liability. This would require robust mechanisms for adjudicating claims fairly and transparently while safeguarding against abuse and frivolous claims. Additionally, challenges may arise in balancing the principles of no-fault liability with existing legal norms and principles of justice.

In the context of India's diverse and unorganized healthcare system, several challenges would need to be addressed for the successful implementation of a no-fault liability system. These include disparities in access to healthcare services, variations in quality of care, and limited awareness among the population about their rights and recourse mechanisms. Moreover, the sheer size and complexity of India's healthcare landscape pose logistical challenges in establishing and managing a nationwide compensation scheme. Ensuring adequate funding, capacity building, and stakeholder engagement would be imperative to overcome these hurdles and ensure the effective functioning of a no-fault liability system in India. It would probably take a minimum of a decade of systematic improvement in legal and insurance systems for India to be in a position to consider adopting this system.

The final verdict

In conclusion, while the concept of implementing a no-fault liability system in India's healthcare sector is intriguing, the current landscape presents several hurdles. Challenges such as disparities in access, varying healthcare practices, and resource constraints highlight the complexity of such a transition. For India to be ready in the future, significant improvements in healthcare infrastructure, stakeholder engagement, and capacity-building efforts are imperative. Addressing these challenges will be crucial in laying the foundation for the potential adoption of a no-fault liability system in India's healthcare system.
